# Effects of tumor necrosis factor-α on glucose uptake in human granulosa cells under high androgen conditions

**DOI:** 10.22038/IJBMS.2023.68784.14993

**Published:** 2023

**Authors:** Dongxia Sun, Yuanyuan Zhao, Xiaohua Wu

**Affiliations:** 1 Hebei Medical University, Shijiazhuang, 050011, China; 2 Prenatal Diagnosis Center, The Fourth Hospital of Shijiazhaung Affiliated to Hebei Medical University, Shijiazhuang 050011, China; 3 Institute of Reproductive Medicine of Shijiazhuang, The Fourth Hospital of Shijiazhuang, Gynecology and Obstetrics Hospital Affiliated to Hebei Medical University, Shijiazhuang 050011, China

**Keywords:** Glucose uptake, Granulosa cells, NF-kappa B signaling – pathway, Obesity, Polycystic ovary syndrome, TNF-α

## Abstract

**Objective(s):**

Hyperandrogenism is a key pathological characteristic of polycystic ovary syndrome (PCOS). Tumor necrosis factor α (TNF-α) is both an adipokine and a chronic inflammatory factor, which has been proven to be involved in the pathologic process of PCOS. This study aimed to determine how TNF-α affects glucose uptake in human granulosa cells in the presence of high testosterone concentration.

**Materials and Methods:**

KGN cell line was treated with testosterone and TNF-α alone or co-culture combination for 24 hr, or starved for 24 hr. Quantitative real-time polymerase chain reaction (qPCR) and western blot were performed to measure glucose transporter type 4 (GLUT4) message RNA (mRNA) and protein expression in treated KGN cells. Glucose uptake and GLUT4 expression were detected by immunofluorescence (IF). Furthermore, western blot was performed to measure the contents in the nuclear factor kappa-B (NF-κB) pathway. Meantime, upon addition of TNF-α receptor II (TNFRII) inhibitor or Inhibitor of nuclear factor kappa-B kinase subunit beta (IKKβ) antagonist to block the TNFRII-IKKβ-NF-κB signaling pathway, the glucose uptake in KGN cells and GLUT4 translocation to cytomembrane were detected by IF, and related proteins in TNFRII-IKKβ-NF-κB were detected by western blot.

**Results:**

The glucose uptake in Testosterone + TNF-α group was lowered significantly, and Total GLUT4 mRNA and proteins were reduced significantly. GLUT4 translocation to cytomembrane was tarnished visibly; concurrently, the phosphorylated proteins in the TNFRII-IKKβ-NF-κB signaling pathway were enhanced significantly. Furthermore, upon addition of TNFRII inhibitor or IKKβ inhibitor to block the TNFRII-IKKβ-NF-κB signaling pathway, the glucose uptake of treated granulosa cells was improved.

**Conclusion:**

TNFRII and IKKβ antagonists may improve glucose uptake in granulosa cells induced by TNF-α by blocking the TNFRII-IKKβ-NF-κB signaling pathway under high androgen conditions.

## Introduction

Polycystic ovary syndrome (PCOS), one of the most common metabolic and endocrine disorders in women of reproductive age, is characterized by hyperandrogenism, obesity, ovulatory infertility, polycystic ovaries, low-grade chronic inflammation, and insulin resistance (IR) (1). In women who are genetically predisposed to the development of PCOS, weight gain and obesity often result in clinical and biochemical manifestations. The majority of women with PCOS (38%–88%) are either overweight or obese (2-4), of which obesity is one of the leading causes of infertility in those individuals (5, 6). Several studies have shown that glucose metabolism disorder in the ovary could explain the ovulatory dysfunction observed in women with PCOS or obesity (7-9). Accordingly, there are close associations between obesity and PCOS. Clinical studies have reported that PCOS patients are generally in a chronic low-grade inflammatory state which can promote the development of PCOS and that obesity is associated with chronic low-grade inflammation (10, 11). Both PCOS patients and obese patients showed low-grade proinflammatory states including increased adipokine TNF-α in serum. Adipokines may be a connective factor between obesity and PCOS (12) possibly due to immune cell infiltration in obese adipose tissue (13), which produces a number of inflammatory factors, including TNF-α(14), interleukin-6 (IL-6), and IL-1β (1). TNF-α is a master proinflammatory cytokine with a pathogenic role in inflammatory disorders (15), which is expressed by granulosa cells (GCs) and involved in regulating ovarian functions (16).

Several researchers found that the level of TNF-α protein in the follicular fluid (FF) of PCOS patients was significantly elevated (17-19). GCs are closely associated with oocytes and the production of female sex hormones, which play key roles in steroidogenesis, oogenesis, folliculogenesis, atresia, and luteinization(20). TNF-α is both an adipokine and a chronic inflammatory factor that has been proven to be involved in the pathologic process of PCOS and is a potential agent in obesity and androgen expression (21-23). This might suggest that TNF-α with hyperandrogenism may be one of the pathological mechanisms of PCOS with obesity. A principal intracellular effector of the canonical TNF-α signaling pathway is nuclear factor κB (NF-κB) (24). Several researchers proved that TNF-α could down-regulate the glucose uptake of endometrium in PCOS patients with obesity (21). This *in vitro* study was designed to evaluate the effect of TNF-α on GCs with hyperandrogenism. In this study, KGN cells were cultured with TNF-α or testosterone or TNF-α and testosterone to examine whether the TNF-α affected glucose uptake in GCs via the classic TNF-α inflammatory pathway (NF-κB signaling pathway) in the presence of high levels of androgen. 

## Materials and Methods

***Cell culture***

The KGN cell line, a human granulosa-like tumor cell line, was provided by Zhao Yuanyuan, PhD. KGN cells were maintained in 6-cm Petri dishes and cultured in DMEM/F12 -Dulbecco’s Modified Eagle medium (DMEM/F12; Gibco.) supplemented with 10% fetal bovine serum (FBS) (EVERY GREEN), penicillin (100U/l), and streptomycin (100 U/l) (Solarbio Life Science), in a humidified incubator (Thermo Fisher Scientific.) containing 5% CO_2_ at 37 ℃.

***Cell culture***

After adhesion, cells were divided into groups for 24 hr culture with 28.84 ng/ml (100 nM) testosterone alone (content of testosterone>98%, Cayman Chemical), 100 ng/ml TNF-α (recombinant human TNF-α: content of TNF-α>98%, PeproTech) alone, or these two reagents for co-culture. Cells were divided into four groups based on cell culture: T group (culture with testosterone), α group (culture with TNF-α), T+α group (co-culture with testosterone and TNF-α), and con group (the blank control group: starved for 24 hr).

***Addition of inhibitors***

1) Except for the con group, the TNFRII antagonist Qiangke (Celgene Biopharma) was administered to the three culture groups (200 ng/ml) for 24 hr. Then, the cells were collected for subsequent analysis. 2) A phosphorylated IKKB competitive antagonist IMD0354: 1151.01 ng/ml(3 µM) (IMD0354 purity 99.73%, Selleck Chemicals) was used to add testosterone and TNF-α for co-culture of GCs for 0 hr, 1 hr, 2 hr, 4 hr, 6 hr, and 12 hr. After IMD0354 preconditioning for 30 min, testosterone and TNF-α were administered for 0 hr, 1 hr, 2 hr, 4 hr, 6 hr, and 12 hr. In the absence of IMD0354 preconditioning, testosterone, and TNF-α were administered for 0 hr, 1 hr, 2 hr, 4 hr, 6 hr, and 12 hr, and then the cells were collected for subsequent analysis.

***Total RNA extraction and quantitative real-time polymerase chain reaction*** ***(qPCR)***

Total RNA was isolated from KGN cells starved for 24 hr (the con group), treated with testosterone or TNF-α alone or in combination for 24 h, extracted with TRIzol reagent (Solarbio Life Sciences), and reverse transcribed into cDNA with HifairII 1st strand cDNA Synthesis SuperMix for qPCR (11123ES60, Yeasen, Shanghai, China). After removing the remaining DNA according to the instructions, the reverse transcription mix was incubated at 42 °C for 2 min, 25 °C for 5 min, 42 ℃ for 30 min, and 85 ℃ for 5 min. The samples were quantified by qPCR with Hieff SYBR green qPCR master mix (11201ES03/08/60, Yeasen, Shanghai, China) on a qTOWER3 G RT–PCR system (Analytic Jena, German) with specific primers, as listed in Table 1. The qPCR conditions were as follows: 95 ℃ for 5 min, followed by 40 cycles at 95 °C for 10 sec and 60 °C for 30 sec. Each sample was examined in triplicate, and each analysis was run three times. The CT values of β-actin were not significantly different among the treatment groups, which confirmed similar sample loading. Relative gene expression was determined by the 2^−^^ΔΔ^CT method, and β-actin was used as a reference gene. 

***Western blotting***

Western blotting was performed to examine the levels of TNF-α receptor II (TNFRII), I-kappa-β kinase (IKKβ), phosphorylated-IKKβ (p-IKKβ), nuclear factor-kappa B p65 (TP65), phosphorylated-NF-κB p65 (p-P65), and glucose transporter-4 (GLUT4). Total protein was extracted by RIPA lysis buffer (Beyotime Institute of Biotechnology) supplemented with complete phosphorylated protease inhibitor cocktail tablets (Servicebio, Wuhan, China) at a 1:100 dilution. Protein concentration was determined by a Nanodrop 2000 (Thermo Fisher, Germany). Equal amounts (quality of WB was 20 ug) of total protein were separated by 10% SDSPAGE and transferred onto 0.22 polyvinylidene difluoride (PVDF) membranes (Millipore, Germany). The membranes were blocked with 5% nonfat milk diluted in 0.01 M Tris-buffered saline supplemented with Tween 20 (TBST) for 1 hr at room temperature (RT) and probed with primary antibodies against TNFRII (1:1000, AF5470, Affinity Biosciences), IKKβ (1:1000, AF6014, Affinity Biosciences), p-IKKβ (1:1,000, AF3009 Affinity Biosciences), TP65 (1:1,000, AF5006, Affinity Biosciences), p-P65 (1:1,000, 3033S, Cell Signaling Technology), GLUT4 (1:1,000 BF1001, Affinity Biosciences), and βactin (1:1,000; GB11001; Servicebio, Wuhan) in 0.01 M TBST with or without 5% nonfat dry milk overnight at 4 °C. Then, the membranes were incubated with horseradish peroxidaseconjugated secondary antibodies at 1:5,000 dilution (anti-mouse, S0002, Affinity Biosciences or anti-rabbit, S0001-100, Affinity Biosciences) for 1 hr at RT. Immunoreactive protein bands were visualized by enhanced chemiluminescence (ECL, BL520A, Biosharp Life Sciences) on a multifunctional imaging system. Blots were scanned and quantified with image analysis software (VILBER LOURMAT FUSION FX7 EDGE). All densities of the target protein band were normalized to β-actin.

***GLUT4 immunofluorescence (IF) analysis***

After treatment, the medium was removed, and the cells were rinsed with 1× PBS for 30 sec at RT, incubated with 2 ml of 4% paraformaldehyde (PFA, P1110, Solarbio Life Sciences) for 10 min at RT, and rinsed 3 times at RT with 1× PBS for 30 sec. Then, 60 µl of primary antibody against GLUT4 (1:200 BF1001, Affinity Biosciences) diluted in 5% normal goat serum (NGS SL038-10, Solarbio Life Sciences) in 1× PBS was applied to the coverslips, which were incubated overnight at 4 ℃. The group without primary antibodies served as the negative control group. The specimens were incubated with 60 µl of secondary antibody (goat-anti-mouse, A23210, Abbkine Scientific Co.) diluted in 5% NGS/1× PBS for 1 hr at RT. Coverslip slides were sealed with one drop of antifading mounting medium (with DAPI) (S2110, Solarbio Life Sciences) for 2 hr at RT. Green fluorescence intensity was measured with an FV-ASW system (Olympus Confocal microscope FV3000, Olympus, Japan). The average grey value was determined with Cellsens.

***Glucose uptake***

After KGN cells were treated, the maintenance medium was replaced with 400 μl of serum-free DMEM/F12 for a 30-min preincubation in a 37 °C incubator. The cells were washed two times with precooled PBS. The fluorescent glucose analog 34226ng/ml (100 μM) 2-NBDG (purity >98%, B6035, APEXBIO) and 580.77 ng/ml (100nM) insulin (purity >98%, I8830, Solarbio Life Sciences) were added and incubated for 1 hr, and then the cells were isolated (for protein and mRNA extraction). 2-NBDG uptake was terminated by removing the medium, and the cells were washed twice with precooled PBS. Some cells were trypsinized and resuspended in 400 μl of precooled PBS, while the remaining cells were stained with DAPI (C0060, Solarbio Life Sciences) and observed with an Olympus confocal microscope (FV3000, Olympus, Japan). The average grey values analysis was conducted using Cellsens.

***Statistical analysis***

The data are presented as the mean±SD. Statistical analysis was carried out by GraphPad Prism (version 8.0.2, GraphPad Software, Inc.). Levene’s test was used to test the normality of data distribution. Differential analysis was conducted by one-way analysis of variance (ANOVA), followed by the *post hoc* Duncan test. SPSS 21.0 (SPSS, USA) was used for data analysis, and *P*<0.05 was considered statistically significant, while *P*<0.01 was considered statistically dramatic.

**Table 1:** Primers used in quantitative Real-time PCR for amplification of GLUT4 and β-actin

Gene	Forward primer (5'‑3')	Reverse primer (5'‑3')
GLUT4	CGACCAGCATCTTCGAGACA	CACCAACAACACCGAGACCA
β-actin	CACCATTGGCAATGAGCGGTTC	AGGTCTTTGCGGATGTCCACGT

GLUT4 glucose transporter type four

**Figure 1:**
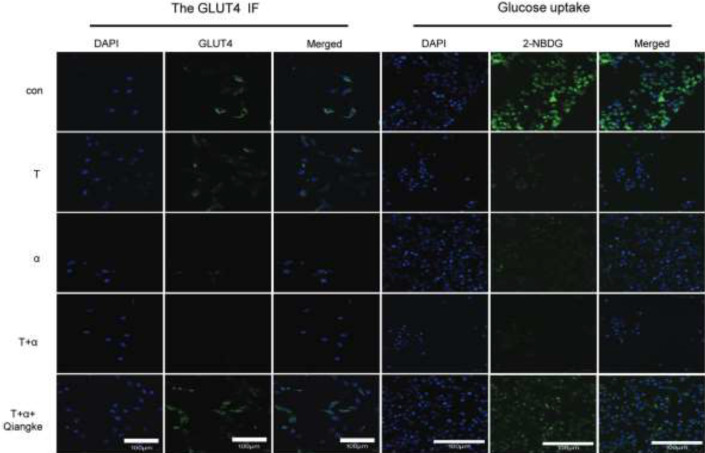
Immunofluorescence images of GLUT4 and glucose uptake in KGN cells

KGN cells were starved for 24 hr and treated with testosterone or TNF-α alone or in combination or testosterone +TNF-α+TNFRII inhibitor (Qiangke) for 24 hr. The left panel detects GLUT4, green shows GLUT4 expression, and blue shows the nucleus. The images of GLUT4 were taken using a confocal microscope (FV3000, Olympus, Tokyo, Japan) with a 10X, 0.4 N.A. objective. Scale bars =100 μm; The right panel measures glucose uptake, with green showing the amount of glucose uptake and blue showing the nucleus. The images of glucose uptake were taken using a confocal microscope (FV3000,

Olympus, Tokyo, Japan) with a 10X, 0.4 N.A. objective. Scale bars =100 μm

**Figure 2:**
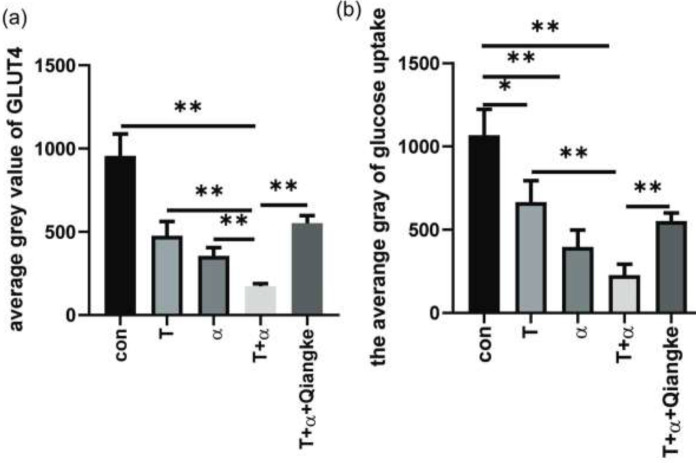
(a) Data of GLUT4 in cytomembrane from KGN cells starved for 24 hr and treated with testosterone or TNF-α alone or testosterone+ TNF-α+ TNFRII inhibitor (Qiangke) for 24 hr vs KGN cells treated with testosterone and TNF-α. (*F*=7.015; *P<0.05, **P<0.01) (b) Data of glucose uptake in KGN cells starved for 24 hr and treated with testosterone or TNF-α alone or testosterone+ TNF-α+ TNFRII inhibitor (Qiangke) for 24 hr vs KGN cells treated with testosterone and TNF-α. (*F*=5.852; *P<0.05, **P<0.01)

**Figure 3:**
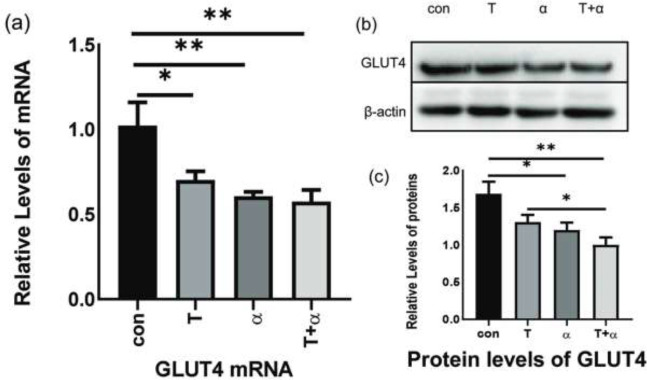
(a) Data of total GLUT4 mRNA from KGN cells starved for 24 hr and treated with testosterone or TNF-α alone or in combination for 24 hr( *F*= 2.194;*P<0.05 **P<0.01). (b) Western blot of total GLUT4 protein expression from KGN cells starved for 24 hr and treated with testosterone or TNF-α alone or in combination for 24 hr. (c) Based on western blot in the image (b), the histogram shows the ratio of the gray value of the target protein GLUT4 to that of β-actin. (*F* =0.189; *P<0.05, **P<0.01)

**Figure 4:**
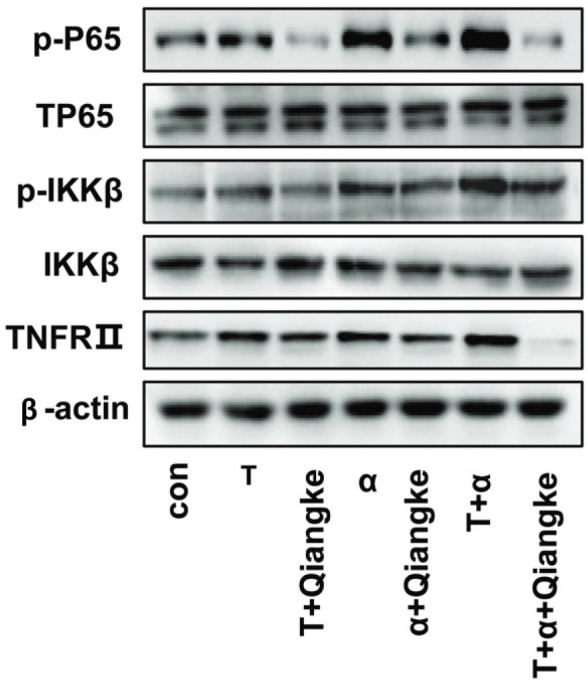
Western blot of p-P65, TP65, p-IKKβ, IKKβ, TNFRII, β-actin protein expression from KGN cells starved for 24 hr and treated with testosterone or testosterone +TNFRII inhibitor (Qiangke) or TNF-α, or TNF-α+TNFRII inhibitor (Qiangke) or testosterone +TNF-α or testosterone +TNF-α+TNFRII inhibitor (Qiangke) for 24 hr

**Figure 5:**
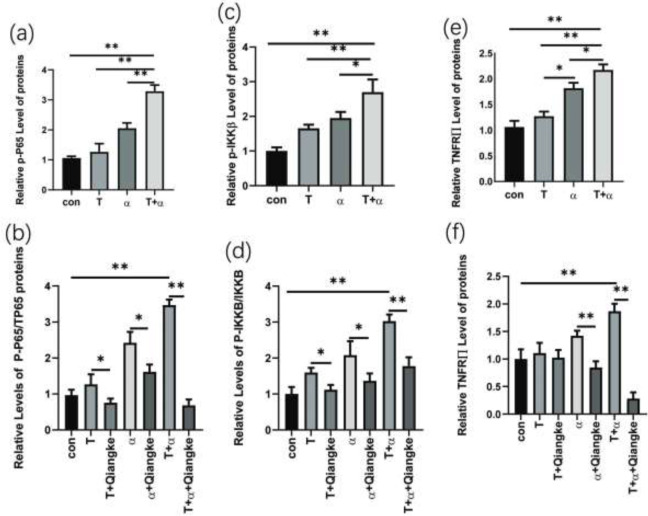
Based on the Western blot in Figure 4, the histogram shows the ratio of the gray value of the target protein to that of β-actin: p-P65 (p-P65/TP65/β-actin), p-IKKβ (p-IKKβ/IKKβ/β-actin), TNFRII (TNFRII/β-actin). (a), (c), (e) Data of p-P65, p-IKKβ, and TNFRII are presented in the KGN cells treated with testosterone or TNF-α alone or in combination. (b), (d), (f) Data of p-P65, p-IKKβ, and TNFRII are presented in the KGN cells treated with testosterone or TNF-α or testosterone +TNF-α and added with TNFRII inhibitor (Qiangke), respectively. (*F*=0.660, 1.153 0.279; *P<0.05; **P<0.01)

**Figure 6:**
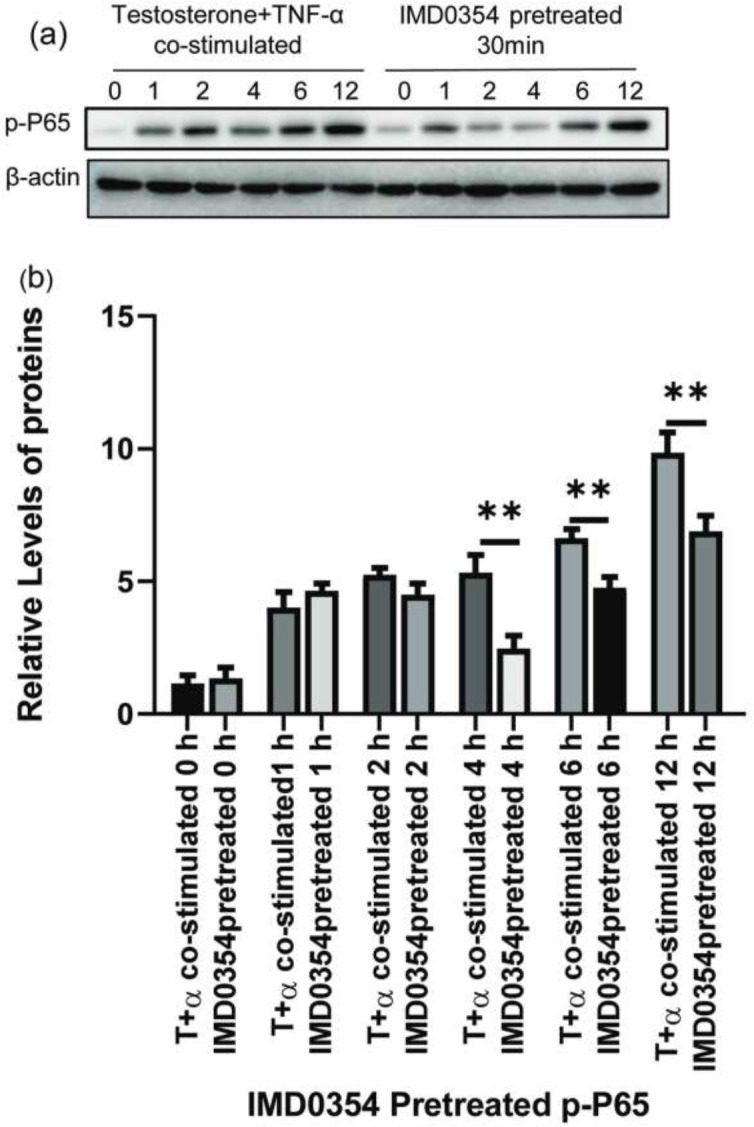
(a) Western blot of p-P65 (p-P65/TP65/β-actin) protein expression from KGN cells pretreated with or without IKKβ inhibitor (IMD0354) for 30 min, which were treated with testosterone +TNF-α for 0 hr, 1 hr, 2 hr, 4 hr, 6 hr, and 12 hr. Data are presented as relative to control that was set as (b). (*F*=0.690;*P<0.05; **P<0.01)

**Figure 7:**
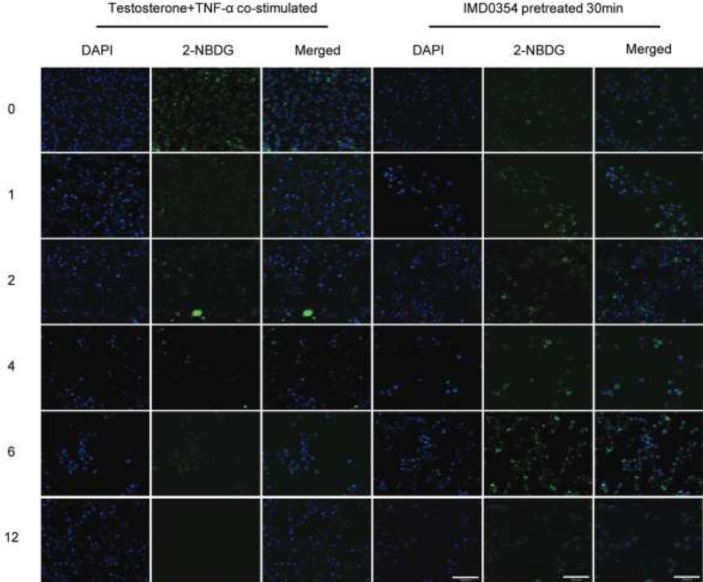
Immunofluorescence images of glucose uptake in KGN cells

The glucose uptake (2-NBDG) from KGN cells pretreated with or without IKKβ inhibitor (IMD0354) for 30 min, which were treated with testosterone +TNF-α for co-stimulation for 0 hr, 1 hr, 2 hr, 4 hr, 6 hr, and 12 hr. The green fluorescence shows the amount of glucose uptake and blue shows the nucleus. Images were taken using a confocal microscope (FV3000, Olympus, Tokyo, Japan) with a 10X, 0.4 N.A. objective. Scale bars =100 μm

**Figure 8:**
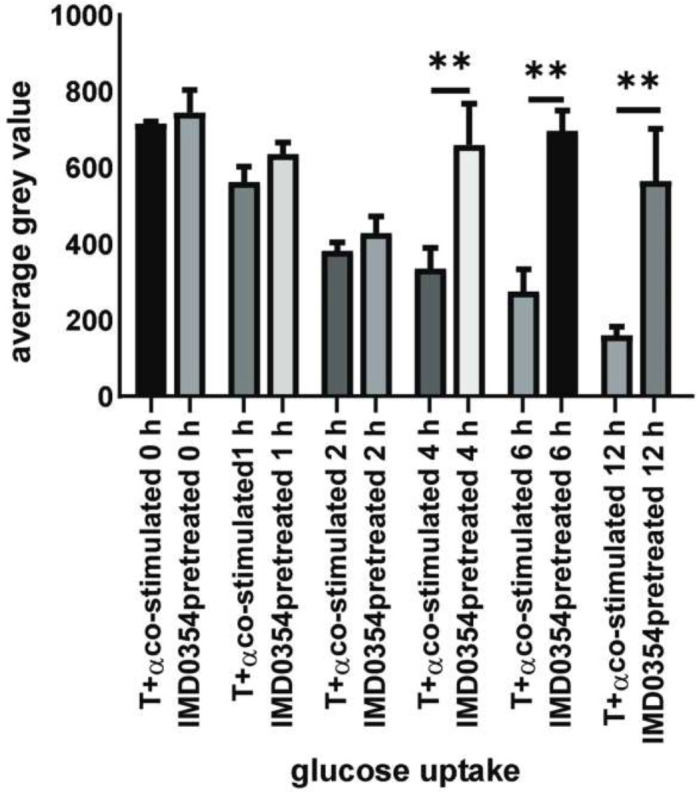
Data of glucos e uptake (2-NBDG) from KGN cells pretreated with or without IKKβ inhibitor (IMD0354) for 30 min, stimulated by testosterone +TNF-α for 0 hr, 1 hr, 2 hr, 4 hr, 6 hr, and 12 hr. (*F*=9.262; *P<0.05; **P<0.01)

## Results

***Glucose uptake of GCs coculture with TNF-α under high testosterone was lowered significantly***

The effects of each treatment on glucose uptake were assessed by a glucose fluorescence substitute (2-NBDG)-based assay. 2-NBDG uptake in KGN cells of the T group decreased significantly compared with the blank control group (Figure 1, Figure 2b, *P*<0.05). 2-NBDG uptake in the groups that were cultured with TNF-α alone or testosterone and TNF-α was drastically decreased compared with the blank control group (Figure 1, Figure 2b, *P*<0.01). In addition, intracellular green fluorescence decreased with cotreatment.

***GLUT4 level of GCs cultured with TNF-α under high testosterone was lowered significantly***

The results showed that the total GLUT4 mRNA and proteins in KGN cells were reduced in the T+α group. Total GLUT4 mRNA level was significantly decreased in the T group, as compared with the con group (Figure 3a, *P*<0.05), as well as in α or T+α groups, as compared with the con group (Figure 3a, *P*<0.01). In the meantime, GlUT4 protein expression was significantly decreased in α group vs con group and T+α group vs T group (Figure 3b, 3c, *P*<0.05). In addition, the expression in T+α group was drastically decreased compared with the con group (Figure 3b, 3c, *P*<0.01). GlUT4 protein is a glucose transporter that is embedded in the cell membrane. When Glut4 migrates from the cytoplasm to the cell membrane, glucose uptake is increased. We measured the GLUT4 immunofluorescence intensity in the cytomembrane after TNF-α or testosterone or TNF-α and testosterone were added to KGN cells. The results showed that when the KGN cell line was cocultured with TNF-α and testosterone, the presentation of GLUT4 on the cytomembrane was gradually reduced (Figure 1 and Figure 2a).

***Proteins level of TNFRII-NF-κB signaling pathway in GCs culture with TNF-α and testosterone***

TNF-α significantly increased TNFRII, P-IKKβ, and p-P65 in the presence of high testosterone compared with TNF-α alone or testosterone alone visibly (Figure 4). TNF-α is a major inducer of the NF-κB signaling pathway. When KGN cells were cultured with TNF-α and testosterone, the levels of TNFRII and P-IKKβ were increased significantly in the T+α group compared with those in the α group (Figure 5c, 5e, *P﹤*0.05), and those expressions were increased drastically in the T+α group compared with those in the T group and the blank control group respectively (Figure 5c, 5e, *P﹤*0.01). p-P65 expression was drastically higher in the T+α group than in the α group, T group, and blank control group (Figure 5a, *P﹤*0.01). These results indicated that the inflammatory pathways induced by TNF-α, the NF-κB signaling pathway, were visibly amplified in a high testosterone environment (Figure 4). 

***Effect of TNFRII blocking on KGN cells cultured with TNF-α and high-level testosterone***

Blocking TNFRII with a TNF-α receptor II inhibitor (Qiangke) on KGN cells cultured with testosterone, TNF-α or cocultured with testosterone and TNF-α, the protein level of TNFRII (TNFRII/β-actin), p-IKKβ (p-IKKβ/ IKKβ/β-actin), and p-P65(p-P65/TP65/β-actin) were all reduced (Figure 4, Figure 5b, c, d, *P*<0.01), especially in the T+α group which was cultured with a TNFRII inhibitor Qiangke. These results suggest that the TNFRII inhibitor could reduce the phosphorylated protein levels of NF-κB signaling pathway factors that were activated by TNF-α, particularly in a high-testosterone environment. In the presence of a TNFRII inhibitor QiangKe, the expression of GLUT4 on the cytomembrane was increased significantly. (Figure 1, Figure 2a; *P*<0.05). Then when a TNFRII inhibitor QiangKe was added, 2-NBDG uptake in treated cells was increased visibly (Figure 1, Figure 2b; *P*<0.01).

***Effect of IKKβ*** ***blocking on KGN cells*** ***cultured with TNF-α and high-level testosterone***

IMD0354, as one of selectivity IKKβ inhibitors, could generate a blocking effect on the NF-κB signaling pathway. After pretreatment with IMD0354 (IKKβ inhibitor) for 30 min, KGN cells were stimulated with TNF-α (100 ng/ml) and testosterone (28.84 ng/ml) for 0 hr, 1 hr, 2 hr, 4 hr, 6 hr, and 12 hr. The level of p-P65 in cells that were pretreated with IMD0354 for 4 hr, 6 hr, and 12 hr was lower than in treated cells, and these results were statistically significant (Figure 6a, b; *P*<0.05). In the presence of a high testosterone environment, inflammatory responses induced by TNF-α could be significantly reduced by pretreating with IKKβ inhibitor. When the cells were pretreated with an IKKβ inhibitor (IMD0354), intracellular green fluorescence was evidently increased compared with that of cells that were treated with TNF-α and testosterone for 4 hr, 6 hr, and 12 hr (Figure 7, Figure 8; *P*<0.01)*.*

## Discussion

PCOS is characterized by hyperandrogenism, obesity, IR, and ovulatory dysfunction. As a heterogeneous hormone-imbalance gynecological disorder, the estimated prevalence of PCOS is approximately 4–21% among adolescents and reproductive-aged women, depending on the definition criteria of PCOS (25, 26). A large number of women with PCOS are also overweight or obese (27). Regarding obesity, it is known that the increase of adipose tissue involves changes in the molecular expression pattern of these cells, altering the secretion of TNF-α (28, 29). We assessed the effect of TNF-α, an important proinflammatory cytokine, on the glucose uptake ability of GCs exposed to a high testosterone environment. Glucose is a hydrophilic molecule that is transported through the cell membrane with glucose transporters. As is well known, glucose availability is vital for human GCs to fulfill normal functions. Consequently, glucose uptake is mediated by a number of facultative sugar transporters (30, 31). In PCOS, IR occurs in the ovaries (32, 33), in addition to other classic target tissues, such as skeletal muscle, fat, and the liver (34, 35). GLUT4 is a high-affinity GLUT that is predominantly expressed in muscle cells and adiposity and mediates insulin-stimulated glucose transport(36). We explored the effect of TNF-α, an important proinflammatory cytokine, on the glucose uptake ability of GCs exposed to a high testosterone environment. Our experiment results showed that the GLUT4 protein and mRNA levels of the cells cultured with testosterone and TNF-α were significantly lower than that of the control cells. Related literature has shown that PCOS patients with obesity were affected by both high androgen and TNF-α levels(37). Considering the experimental results and clinical features of PCOS, this study explored its possible mechanism.

TNF-α exerts a wide range of biological activities and is a “master regulator” of immune and inflammatory responses(38). A high percentage of women with PCOS exhibit hyperinsulinemia and obesity (37). Several studies have verified that the TNF-α level was higher in the serum and follicular fluid (when collected at oocyte retrieval) of women with simple obesity and PCOS patients with obesity (39). Our experimental results displayed that both testosterone and TNF-α co-cultured the KGN cell line, resulting in significantly increased p-IKKβ and p-P65, thus suggesting that TNF-α highly activated the NF-κB signaling pathway in a high testosterone environment. To some extent, this indicates the proinflammatory station of PCOS patients with obesity is more severe. When a TNFRII receptor inhibitor (Qiangke) was added, p-IKKβ and p-P65 protein levels were all reduced in T, α, and T+α groups, but these phosphorylated levels of proteins were significantly lower in T and T+α groups. In addition, GLUT4 translocation and glucose uptake by cells were determined using IF, and GLUT 4 levels in the cytomembrane and the glucose uptake by cells were remarkably lower than those of untreated cells. Despite no significant difference in total mRNA and GLUT4 protein levels between the α and T+α groups, significant differences were found in glucose uptake and GLUT4 in cytomembrane as measured by IF. When the cells were pretreated with an IKKβ inhibitor (IMD0354), p-IKKβ and p-P65 protein levels were lowered by testosterone and TNF-α (30 min, 1 hr, 4 hr, and 6 hr), and GLUT4 translocation to the cell membrane and glucose uptake by KGN cells were better than those of cells treated with testosterone or TNF-α, especially those treated with testosterone and TNF-α. Our experimental results showed that GLUT4 and glucose uptake were simultaneously rescued after one of the important target proteins of the NF-κB pathway was blocked by the inhibitor.

The experimental results indicated that the glucose uptake of GCs could be regulated via TNF-α-TNFRII-IKKB-NF-κBp65, the major TNF-α signaling pathway. Chronic inflammation is a typical characteristic of PCOS, in which TNF-α plays a pivotal role (22). TNF-α has aroused great attention in a variety of diseases, and numerous studies have confirmed its roles in physiological activities (22, 40, 41), which prompted us to investigate the use of TNF-α as a therapeutic agent against the pathogenesis of PCOS. In the current study, TNF-α could decrease the glucose uptake in GCs through the TNFRII-IKKB-NF-κB signaling pathway in high concentrations of testosterone. Meanwhile, TNFRII inhibitor and IKKB inhibitor could improve the glucose uptake in GCs.

## Conclusion

Our experimental results showed that highly increased TNF-α might be an important factor leading to the dysfunction of ovarian GCs, especially in the presence of pathologically enhanced androgen. In the meantime, we attempted to use anti-TNF-α to improve the function of GCs. Our results also revealed that anti-TNF-α or blocking the NF-κB pathway could increase the glucose uptake in GCs. However, this study has several limitations. It included a relatively small number of experiments on other cell lines and healthy control cell lines. Therefore, additional experiments need to be performed on other cell lines.

## Authors’ Contributions

DX S and XH W designed the experiments; DX S performed experiments and collected data; DX S and XH W discussed the results and strategy; XH W supervised, directed, and managed the study; DX S and XH W approved the final version to be published.

## Funding

This study was supported by the Natural Science Foundation of Hebei Province (Grant No. H2019106051) and the project of Shijiazhuang Science and Technology Bureau (Study on the effect and mechanism of human umbilical cord mesenchymal stem cell-derived exosomes on chronic inflammation of PCOS) (No.211460513).

## Availability of Data and Materials

The datasets used and/or analyzed during the current study are available from the corresponding author upon reasonable request.

## Conflicts of Interest

The authors declare that they have no competing interests.
